# Internalization of *Lactobacillus crispatus* Through Caveolin-1-Mediated Endocytosis Boosts Cellular Uptake but Blocks the Transcellular Passage of *Neisseria meningitidis*

**DOI:** 10.3390/microorganisms13030479

**Published:** 2025-02-21

**Authors:** Kenny Lidberg, Sarah Pilheden, Mikel Relloso Ortiz de Uriarte, Ann-Beth Jonsson

**Affiliations:** Department of Molecular Biosciences, The Wenner-Gren Institute, Stockholm University, 106 91 Stockholm, Sweden; kenny.lidberg@su.se (K.L.); sarah.pilheden@su.se (S.P.); mikelrelub@gmail.com (M.R.O.d.U.)

**Keywords:** *Lactobacillus*, *Neisseria meningitidis*, invasion, caveolin-mediated endocytosis, pathogenic clearance

## Abstract

*Neisseria meningitidis* is a human-specific pathogen that colonizes the nasopharyngeal epithelium, which is populated by a dynamic microbiota that includes *Lactobacillus* species. Currently, little is known about the interaction between commensal lactobacilli and pathogenic *Neisseria*, emphasizing a need for deeper studies into the molecular interactions between the two bacteria species. This, in turn, could add clinical and therapeutic value to existing treatments against an *N. meningitidis* infection. In this work, we explored how lactobacilli affect the interplay between *N. meningitidis* and host cells. We report that *Lactobacillus crispatus*, but not other tested *Lactobacillus* species, efficiently enters pharyngeal cells via caveolin-mediated lipid raft endocytosis and simultaneously enhances the uptake of *N. meningitidis*, as well as uptake of other pathogenic and non-pathogenic microbes. After promoting internalization, *L. crispatus* then prevented *N. meningitidis* from being released and transcytozed from a confluent cell layer on microporous transwell membranes. Infected cells increased the level of acidic vacuoles and pathogen clearance over time, while lactobacilli survived inside the cells. Taken together, the data suggest a possible route through which the cellular uptake of lactobacilli can increase the uptake of pathogens for destruction.

## 1. Introduction

The obligate human opportunistic pathogen *Neisseria meningitidis* (meningococcus) can colonize the nasopharyngeal mucosa and reside there asymptomatically. About 10–35% of the human population carry this bacterium as part of their normal flora [1]. However, for reasons not yet fully understood, it can occasionally disseminate into the tissue and bloodstream and cause severe diseases such as bacterial sepsis and inflammation of the meninges [2]. *N. meningitidis* can be transmitted through the nasopharyngeal mucosa of its host via aerosol droplets, where it uses multiple strategies and virulence factors for efficient colonization [3].

The bacterium initially uses Type IV pili (tfp) to attach to the cells so that they can avoid being swept away [4]. *N. meningitidis* is an epicellular bacteria that resides in the mucosa or on the cell surface, but it can also invade and transmigrate to other areas. Opacity proteins (Opas) mediate the invasion of host cells by binding to carcinoembryonic antigen-related cell adhesion molecules (CEACAMs), which triggers endocytosis of the bacteria into epithelial cells and transcytosis of microorganisms through intact epithelial layers [5]. In this intimate attachment and possible invasion, the meningococcal capsule has to be downregulated via the contact-regulated gene A (CrgA) regulator [6]. Meningococcal lipopolysaccharide (LPS) is necessary for close attachment and invasiveness, although it is less crucial than the CEACAM-Opa mechanism [7]. As an epicellular bacteria, *N. meningitids* has additional mechanisms to survive inside the cell, adopting a facultative intracellular lifestyle before sequestering GTPases from the Rab-family that regulates and controls the endocytosis and exocytosis pathways, leading to the sequestering of lactoferrin and changing of cell polarity; with the loss of polarity, *N. meningitidis* can exit across the basolateral surfaces [8].

The commensal nasopharyngeal microbiota includes *Lactobacillus* spp., which are most abundant in younger children who are born vaginally, and the most common species are the vaginal isolates *L. crispatus* and *L. gasseri* [9]. *Lactobacillus* species are known to have health-promoting mechanisms for the host. Some of these mechanisms act directly against an invading pathogen by out-competing them for nutrients and space by using their surface-layered proteins (SLPs) to bind to host collagen or fibronectin [10,11]. Other mechanisms involve the release of antimicrobial substances such as organic acids, bacteriocins, or biosurfactants [12]. Additionally, lactobacilli can influence the host directly by promoting increased mucosal thickness, tightness between cells, immune system maturation, or increased cell proliferation [13,14]. In turn, the host can promote colonization by lactobacilli through upregulation of receptors or by releasing nutrients aimed at promoting the healthy growth of probiotic bacteria [15].

Endocytic pathways, including F-actin-mediated phagocytosis/micropinocytosis, clathrin/receptor-mediated endocytosis, and caveolin/lipid rafts-mediated endocytosis, are the main molecular mechanisms that have been described for extracellular uptake of material such as nutrients, immunoregulatory molecules, or bacteria [16,17]. As non-professional phagocytic cells, epithelial cells usually engulf bacteria to a very low degree compared to professional phagocytes. However, the accumulative effect of bacteria being killed by epithelial cells does contribute to microbial clearance [18]. Internalization of bacteria can function as a way to clear bacteria and kill them, similar to phagocytosis, via lysosome-mediated destruction or autophagy, but it can similarly be exploited by bacteria to invade the cells [19,20]. Until recently, lactobacilli were not thought to be internalized by epithelial cells, but observations of *Lacticaseibacillus paracasei* and *Lacticaseibacillus rhamnosus* have shown that they are capable of entering intestinal epithelial cells and surviving intracellularly without causing harm to the host [21].

In this study, we investigated how different *Lactobacillus* strains affect the interaction between *N. meningitidis* and host pharyngeal cells. None of the tested lactobacilli inhibited meningococcal adherence to epithelial cells. Instead, the presence of *L. crispatus* increased meningococcal internalization into host epithelial cells. Interestingly, *L. crispatus* itself was efficiently internalized via caveolin-mediated endocytosis. Internalized *L. crispatus* survived inside host cells, whereas *N. meningitidis* was killed over time. These findings shed light on how the composition of the microflora can interfere with meningococcal uptake and release by target epithelial cells, highlighting a potential role for *L. crispatus* in modulating host–pathogen interactions.

## 2. Materials and Methods

### 2.1. Bacterial Strain and Growth Conditions

All of the bacteria used in this study are listed in Supplementary Materials Table S1. *Neisseria meningitidis* FAM20, a serogroup C strain, and JB515, a serogroup W strain, have been described previously [22]. The pathogenic *Neisseria* was grown on GC agar (Neugen Cultured Media, Lansing, MI, USA) supplemented with 1% Kellogg’s solution [23] for 16–18 h at 37 °C in 5% CO_2_ and a humidified environment. Four bacteria previously belonging to the genus *Lactobacillus* before the nomenclature change in March 2020 [24] are still collectively called lactobacilli in this paper. These bacteria were isolated from healthy human donors and have been described previously [25,26].

The lactobacilli were inoculated into liquid MRS medium (Oxoid Inc, Hampshire, UK) for 16–18 h in the same conditions as *N. meningitidis*. On the day of the experiment, all bacteria were added to a liquid medium and grown into the log phase. *N. meningitidis* was inoculated into liquid GC media supplemented with 1% Kellogg’s solution and an overnight culture of lactobacilli, which was diluted to 1:5 in a new fresh MRS medium. *E. coli* O111:B4 [27] was grown on LB liquid culture and LB-agar plates in similar environmental conditions as *Neisseria*.

### 2.2. Cell Lines and Growth Conditions

The human epithelial squamous carcinoma cell line FaDu [28] (ATCC HTB-43) was maintained in Dulbecco’s modified Eagle’s medium containing GlutaMax and pyruvate (DMEM; Thermo Fisher Scientific, Waltham, MA, USA) and supplemented with 10% heat-inactivated fetal bovine serum (FBS; Sigma-Aldrich, Burlington MA, USA). Primary Human Nasal Epithelial Cells (HNEpCs) (PromoCell, Heidelberg, Germany), isolated from normal human nasal mucosa, were grown in supplemented media according to the supplier’s recommendations. Cells were maintained at 37 °C and 5% CO_2_ in a humidified environment. In a 24-well plate, FaDu cells were grown to confluency, HNEpC cells were grown to 50% confluency, and they were washed before the experiment.

### 2.3. Adherence Assay

Log-phase *N. meningitidis* and lactobacilli were pelleted by centrifugation and resuspended in DMEM supplemented with 1% FBS and 1 mM sodium L-lactate (Sigma-Aldrich), which was the experimental media used for all assays. FaDu cells were infected with bacteria at a multiplicity of infection (MOI) of 100 and at a ratio of 1:1 when co-incubating two strains. In adherence assays, lactobacilli were incubated with cells for 1 h before washing 2 times in PBS and the addition of *N. meningitidis*. After incubation for 2 h, unbound bacteria were removed by washing, and cells were lysed with 1% saponin in DMEM. Adherence was estimated by a viable count with GC agar for *N. meningitidis* and solid Rogosa agar plates (Oxoid Inc.) for lactobacilli. Raw data of bacterial counts are presented in Supplementary Materials Table S2.

### 2.4. Gentamicin Invasion Assay

Log-phase bacteria were pelleted, washed, and resuspended in DMEM. Host cells (FaDu or HNEpC) were washed and inoculated at MOI 100 for each bacterial strain as a single species or co-culture. Cells were seeded in 24-well plates before each experiment. In co-culture, *Lactobacillus* was added first for 1 h before the addition of *N. meningitidis*. After 6 h of incubation, 200 µg/mL of sodium gentamicin (Sigma Aldrich) was added for 1 h. After this step, cells were washed and treated with 1% saponinand plated on GC, Rogosa, or LB agar plates to enumerate internalized viable *N. meningitidis,* lactobacilli, or *E. coli,* respectively. In the experiment where the media was acidified, lactic acid (Sigma-Aldrich) was used.

### 2.5. Flow Cytometry Invasion Assay

The invasion of bacteria was quantified by a flow cytometry assay [29,30]. Log-phase bacteria were pelleted, washed, and resuspended in DMEM. FaDu cells in 24 well plates were washed and inoculated with MOI 100 of each bacterial strain as a single species or in co-culture. In co-culture, *Lactobacillus* was added first for 1 h before infection with *N. meningitidis*. During the incubation with lactobacilli, *N. meningitidis* was resuspended to 10^8^ CFU/mL and stained with 5-(6)-carboxyfluorescein-succinimidyl ester (CFSE; Molecular Probes, Eugene, OR) for 15 min. The staining was quenched with an equal volume of FBS for 15 min, after which a washing step was performed, and the pellet was resuspended in DMEM. Labeled *N. meningitidis* was then added to cells for 6 h alone or in lactobacilli co-culture. Post-incubation cells were trypsinized in 0.05% trypsin-EDTA (Thermo Fisher Scientific), washed in PBS, and fixed in 4% methanol-free paraformaldehyde (PFA, Sigma-Aldrich) for 15 min.

The flow analysis was performed with an LSRFortessa flow cytometer (BD Biosciences, Frankling Lakes, NJ, USA). Before counting, the uninternalized bacteria were quenched in 0.2% Trypan blue (Thermo Fisher Scientific) for one minute to be run immediately after. For each sample, 10,000 cells were counted. Data were analyzed with FlowJo software 10.8.2. In experiments using fixed *N. meningitidis*, 4% paraformaldehyde (PFA) was used for 15 min after staining. In experiments to inhibit direct contact between lactobacilli and cells, PET membrane Transwells inserts (6.5 mm diameter; 0.4 µm pore size; Millicell, Sigma-Aldrich, Burlington, MA, USA) were used to separate cells and *N. meningitidis* from lactobacilli.

### 2.6. Fluorescence Microscopy to Detect Intracellular Bacteria

Fluorescence microscopy was performed using a double-stain approach that was previously adopted by Pils et al. [29]. *N. meningitidis* and *L. crispatus* were pre-stained with Sulfo-NHS-LC-Biotin and CellTrace^TM^ CFSE (lactobacilli) or CellTrace^TM^ Far Red (meningococci) (Thermo Fisher Scientific). FaDu cells grown overnight on poly-d-lysine-coated 24-well glass plates (MatTek, Ashland, MA, Wilmington, DE, USA) were pre-treated with MOI 100 of *L. crispatus* and then incubated with *N. meningitidis*. After 6 h of incubation, cells were washed and stained for 45 min with Streptavidin-Alexa 568 to detect possible bacteria that had not been internalized. After staining, cells were fixed with 4% PFA and analyzed using confocal and wide-field microscopy (Zeiss LSM 800 with Airy Scan and Zeiss Widefield Axio Observer 7, Carl Zeiss AB, Oberkochen, Germany). Both *N. meningitidis* and *L. crispatus* were double-stained and imaged together or alone. Uncropped versions of the images can be found in the Supplementary Materials (Figure S1).

### 2.7. CEACAM Detection

Log-phase bacteria were pelleted, washed, resuspended in DMEM, and added to FaDu cells at MOI 100 as a single species for 4 h. For protein expression, the CEACAM expression was quantified with a flow cytometer containing PE-conjugated monoclonal CEACAM antibody (CD66a-B1.1, # 12-0668-42, Thermo Fisher Scientific). The flow analysis was performed with an LSRFortessa flow cytometer (BD Biosciences). In total, 10,000 cells were counted for each sample, and data were analyzed with FlowJo software 10.8.2.

### 2.8. Expression of Virulence Factors

An experiment was performed using the previously described adherence assay with 4 h incubation. RNA was extracted using the RNeasy Plus Mini kit (Qiagen, Venlo, The Netherlands) following the manufacturer’s protocol. The RNA yield and purity were assessed using a NanoDrop 8000, and the RNA was reverse transcribed to cDNA using the SuperScript VILO Mastermix (Thermo Fisher Scientific). The resulting cDNA was amplified using LigthCycler 480 SYBR Green I master mix (Roche, Basel, Switzerland) in a LigthCycler 480 Real-Time PCR system and using the primers listed in Table S3. Gene expression was normalized against the meningococcal housekeeping gene *gdh*.

### 2.9. Internalization Blocking Assay

The method for the internalization-blocking assay was adapted from similar experiments with different bacteria and chemical inhibitors to block internalization using pharmacological inhibitors against the endocytic pathway [16,31,32,33]. This was performed with the same protocol as the gentamicin invasion assay except for the addition of chemical treatments 45 min before the bacteria. The following concentrations were used: dynasore (120 µM), chlorpromazine (45 µM), amiloride (1000 µM), cytochalasin D (2.5 µM), metyl-ß -and cyclodextrin (2500 µM), and nyastin (50 µM) (all chemicals from Sigma-Aldrich).

Caveolin-mediated endocytosis was inhibited by the selective disruptor of caveolin-1 oligomers WL47 (MedChemExpress, Monmouth Junction, NJ, USA) and the caveolin-1 blocking peptide (GTX89541-PEP, Bio-Connect) as described previously [34]. Cells were pre-incubated with the caveolin-1 inhibitors for 30 min before inoculation with bacteria and were kept there during the whole invasion assay. The concentrations used were 50 and 100 nM of WL47 and 1.5 and 15 µg of the peptide. After treatment, cells were visually inspected for morphological changes.

### 2.10. Fluorescence Microscopy to Detect Host Cell Components

Fluorescence microscopy was used to visualize the lipid raft-associated molecules caveolin-1, flotillin-1, and cholesterol. The experimental setup was similar to that used in the gentamicin internalization assay but without the gentamicin step and involved a 24-well glass-bottom plate (MatTek). After 6 h of incubation, cells were washed and stained with polyclonal antibody at 1:5000 against caveolin-1 and flotillin-1 with secondary antibody at 1:5000 conjugated with Alexa-488 or Alexa-594 (Thermo Fisher Scientific). Cholesterol was detected using filipin at 50 µg/mL (Sigma-Aldrich) as reported previously [35].

Acidic vacuoles were visualized with two different stains, one with LysoTracker™ Red DND-99 (LysoTracker, Invitrogen, Carlsbad, CA, USA) and the second with the acridine orange method, previously described [36] with some modifications. The initial procedure for both experiments was the same. Bacteria were inoculated and incubated for 6 h, 22 h, and 30 h, followed by adding gentamicin as described above. For the LysoTracker experiment, the cells were washed in PBS and stained with 0.5 µM LysoTracker and 5 µM Hoechst 33342 (Thermo Fisher Scientific) for 30 min in accordance with the manufacturer’s recommendations. After a PBS wash, the cells were fixed in 4% PFA and imaged with the confocal microscope Zeiss LSM 800 with Airy Scan. For the acridine orange setup, cells were imaged alive after staining for 10 min with 1 µL/mL of the chemical followed directly by microscopy imaging with a Zeiss Widefield Axio Observer 7 microscope (Carl Zeiss AB, Oberkochen, Germany).

### 2.11. Transcytosis

Cells were seeded in PET membrane Transwells inserts (Millicell, Sigma-Aldrich) (6.5 mm diameter; 5 µm pore size) at a concentration of $1.5\times 10^{5}$/well. On every second day, the media was changed to fresh media for 6–7 days [37]. Cells were washed and lactobacilli were added to the upper compartment at an MOI 100 for 1 h. *N. meningitidis* was added at a ratio of 1:1 for co-incubation or alone as a control. According to the manufacturer’s protocol, Lucifer Yellow (LY) (Sigma-Aldrich) was added as a paracellular permeability control at a concentration of 150 µg/mL. After 6 h of incubation, samples were taken from the bottom compartment and plated for the viable count for *N. meningitidis* and lactobacilli. The LY fluorescence absorbance was measured with a SpectraMax i3x microplate reader at 425 nm. *Trans-epithelial* electrical *resistance* (TEER) was measured before adding bacteria and after the 6 h time point [21].

### 2.12. Long-Term Survival Inside Cells

A gentamicin invasion assay was performed at three different time points to test the survivability of the internalized bacteria. Three sets of seeded and inoculated 24-well plates were prepared as in the method for the gentamicin invasion assay with one plate per time point. Time zero in this experiment started after 6 h of incubation plus 1 h of gentamicin treatment. A new medium was added to the plates for the later incubation time points.

After the incubation, *N. meningitidis* and lactobacilli were treated with 1% saponin for 10 min and plated on GC or Rogosa agar, respectively. For testing cell viability in infected cells, an aqua Live/dead™ (Thermo Fisher Scientific) flow cytometry experiment was performed at 6, 22, and 30 h, according to the manufacturer’s protocol. The flow cytometry analysis was performed with an LSRFortessa flow cytometer (BD Biosciences). For each sample, 10,000 cells were counted. Data were analyzed with FlowJo software 10.8.2.

### 2.13. Statistics

Differences between the two groups were analyzed using a two-tailed and unpaired Student T-test. An analysis of variance (ANOVA) was used to analyze differences between multiple groups, followed by a Bonferroni post hoc test. The analyses were performed using GraphPad Prism, version 9.4.1, and *p*-values below 0.05 were considered statistically significant.

## 3. Results

### 3.1. Increased Internalization of Neisseria Meningitidis into Human Epithelial Cells in the Presence of Lactobacillus crispatus

**Figure 1:**
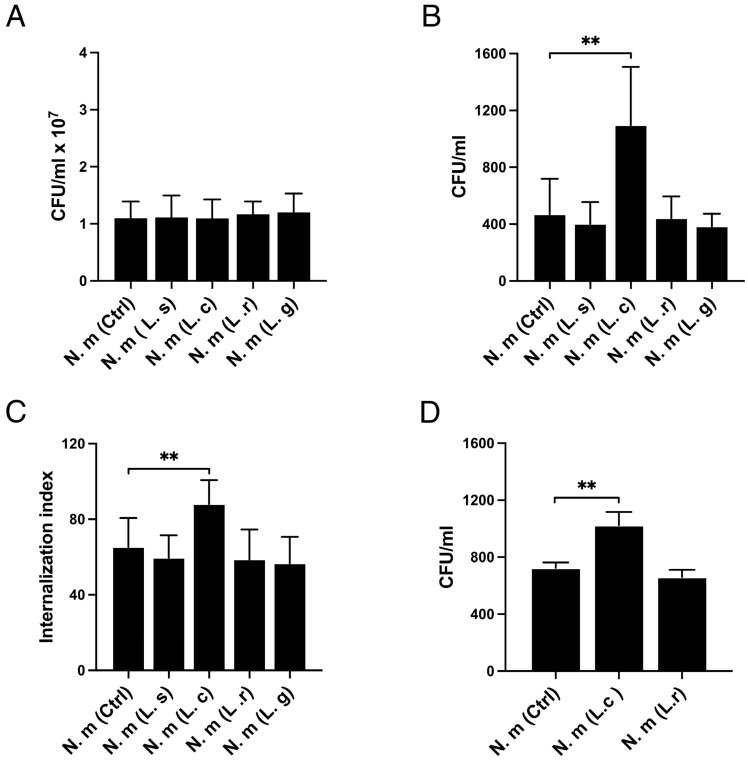
Increased internalization of *Neisseria meningitidis* into human epithelial cells in the presence of *Lactobacillus crispatus.* (**A**) Attachment to FaDu pharyngeal cells of *N. meningitidis* (N. m) in the presence or absence of lactobacilli. Host cells were pre-incubated with *L. salivarius* (L. s), *L. crispatus* (L. c), *L. reuteri* (L. r), *L. gasseri* (L. g), or with medium alone (Ctrl) for 1 h and then infected with *N. meningitidis* for 2 h. Bound bacteria were plated to determine the colony-forming units per mL (CFU/mL). (**B**) Invasion of *N. meningitidis* (N. m) into FaDu cells using the gentamicin assay. Host cells were pre-incubated with lactobacilli or with medium alone (Ctrl) for 1 h, infected with *N. meningitidis* for 6 h, and treated with gentamicin for 1 h to kill extracellular bacteria. Intracellular bacteria were plated for CFU/mL. (**C**) Invasion of *N. meningitidis* into FaDu cells was measured by flow cytometry. CellTrace^TM^ 5-(6)-carboxyfluorescein-succinimidylester (CFSE)-stained *N. meningitidis* was added to cells pre-incubated for 1 h with lactobacilli or with medium alone (Ctrl). Extracellular bacteria were quenched by adding 0.2% Trypan blue before reading. Cells were gated as intact single cells, and 10,000 events were counted. Values are shown as a quantitative internalization index where the percentage of cells with a signal is multiplied by the mean MFI. (**D**) Invasion of Human Nasal Epithelial Cells (HNEpC) by *N. meningitidis* according to the gentamicin assay. Primary epithelial cells were pre-incubated with lactobacilli for 1 h, infected with *N. meningitidis* for 6 h, and treated with gentamicin for 1 h to kill extracellular bacteria. Intracellular bacteria were plated for CFU/mL. Multiplicity of infection (MOI) 100 was used in all experiments. Data represent the mean ± SD of three independent experiments in duplicate. ** *p* < 0.01; unmarked bars are considered non-significant.

Lactobacilli have multiple anti-pathogenic effects against various pathogens and are inhabitants at epithelial surfaces. We investigated whether lactobacilli of different origins could affect colonization and internalization of the opportunistic nasopharyngeal pathogen *N. meningitidis*. Lactobacilli of oral (*L. salivarius*, *L. reuteri*) or vaginal (*L. crispatus*, *L. gasseri*) origin were individually pre-incubated with human pharyngeal FaDu cells for 1 h before infection with *N. meningitidis* for 2 h. The presence of lactobacilli did not affect the attachment of *N. meningitidis* (Figure 1A). *N. meningitidis* alone or co-incubated with *L. crispatus*, *L. salivarius*, *L. reuteri*, or *L. gasseri* adhered equally well.

Next, we examined whether the lactobacilli could affect the invasion of host cells by *N. meningitidis*. Interestingly, the internalization of *N. meningitidis* increased when co-incubated with *L. crispatus* but not when co-incubated with the other *Lactobacillus* strains. This effect was explored in two different quantitative invasion assays, a gentamicin assay (Figure 1B) and a flow cytometry assay (Figure 1C), which both showed that *L. crispatus* increased internalization of *N. meningitidis*, whereas *L. salivarius*, *L. reuteri*, and *L. gasseri* did not. The invasion assays were performed at a 6 h time points. In a control experiment, we showed that the viability of *N. meningitidis* was unaffected by the presence and absence of the lactobacilli (Figure S2). To confirm the findings, we measured meningococcal invasion in an additional cell line, the primary nasal epithelial cell line HNEpC. Similar to the immortalized FaDu cells, internalization of *N. meningitidis* into HNEpC increased when it was co-incubated with *L. crispatus* but not when co-incubated with the control bacteria *L. reuteri* (Figure 1D). Together, the data suggest that the presence of *L. crispatus* can increase the internalization of *N. meningitidis* into epithelial cells.

### 3.2. L. crispatus Must Be in Contact with Host Cells to Increase the Uptake of Both Live and Fixed N. meningitidis

**Figure 2:**
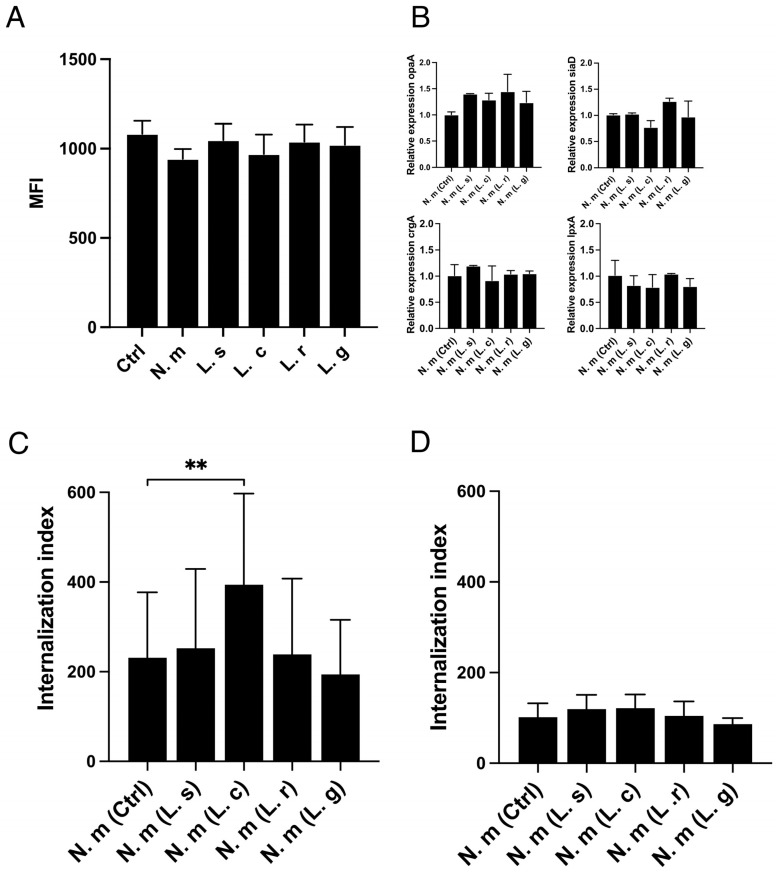
*L. crispatus* must be in contact with host cells to increase uptake of both live and fixed *N. meningitidis.* (**A**) Flow cytometry analysis of CEACAM protein expression in FaDu cells after 4 h of incubation with *N. meningitidis* (N. m), *L. salivarius* (L. s), *L crispatus* (L. c), *L. reuteri* (L. r), or *L. gasseri* (L. g). Untreated cells (Ctrl) were used as the control. A monoclonal PE-conjugated CEACAM antibody was used. CEACAM detection is shown as mean fluorescent intensity (MFI). Data represent the mean of three independent experiments in triplicate. (**B**) Expression of meningococcal invasion-associated genes, *opa, crgA, lpxA*, and *siaD* in the presence of lactobacilli using qPCR. Host cells were pre-incubated with lactobacilli or with medium alone for 1 h before infection with *N. meningitidis* for 4 h. Data represent the mean ± SD of two independent experiments in duplicate and are presented as the fold change relative to uninfected cells. (**C**,**D**) Internalization of *N. meningitidis* (N. m) into FaDu cells either alone or co-incubated with *L. salivarius* (L. s), *L crispatus* (L. c), *L. reuteri* (L. r), or *L. gasseri* (L. g) measured by flow cytometry. (**C**) Internalization of fixed *N. meningitidis* into FaDu cells. Lactobacilli was added 1 h prior to *N. meningitidis*. *N. meningitidis* was CFSE-stained and fixed using 4% PFA before addition to cells for 6 h. (**D**) Invasion of *N. meningitidis* when lactobacilli were separated from host cells in a Transwell culture insert with 0.4 μm pore size; only released molecules could affect the internalization of *N. meningitidis*. FaDu cells were pre-incubated with lactobacilli or medium alone before infection with CFSE-stained bacteria for 6 h. Extracellular bacteria were quenched by 0.2% Trypan blue before reading. Cells were gated as intact single cells, and 10,000 events were counted. Values are shown as the internalization index, where the percentage of cells with a signal is multiplied by the mean MFI, shown as quantitative graphs. Data represent the mean ± SD of three independent experiments in duplicate. ** *p* < 0.01 unmarked bars are considered nonsignificant.

We next investigated potential mechanisms by which *Lactobacillus crispatus* might enhance meningococcal invasion. We performed experiments to study the upregulation of host cell CEACAM receptors, changes in bacterial virulence gene expression, the role of live/dead meningococci, and physical contact between *L. crispatus* and *N. meningitidis*. One of the primary pathways for meningococcal invasion is via Opa-CEACAM binding. However, CEACAM expression on host cells remained unchanged during the co-incubation of meningococci with lactobacilli (Figure 2A). We then analyzed the expression of four genes critical for close contact during invasion: *crgA* (master regulator), *opa* (invasion-related), *lpxA* (first step LPS biosynthesis), and *siaD* (capsule expression). The expression of these genes was similarly unaffected by lactobacilli (Figure 2B). Given the absence of *Lactobacillus*-mediated effects on CEACAM expression and bacterial virulence genes, we explored whether *L. crispatus* could influence the invasion of fixed *N. meningitidis* using quantitative flow cytometry. Remarkably, the internalization of fixed meningococci increased significantly in the presence of live *L. crispatus*, mimicking the effects observed with live meningococci. However, this effect was not observed with the other three lactobacilli strains tested (Figure 2C).

To determine whether direct contact between lactobacilli and host cells was required, we used transwell inserts to physically separate lactobacilli from the cell layer while allowing molecular diffusion. Under these conditions, *L. crispatus* failed to promote meningococcal invasion, indicating that direct contact between *L. crispatus* and host cells is essential for its effect on pathogen uptake (Figure 2D). Taken together, these findings demonstrate that *L. crispatus* can enhance the internalization of both live and fixed meningococci and that direct physical contact with host cells is necessary for this effect.

### 3.3. L. crispatus Enhances the Epithelial Uptake of Both Pathogenic and Commensal Bacteria

**Figure 3:**
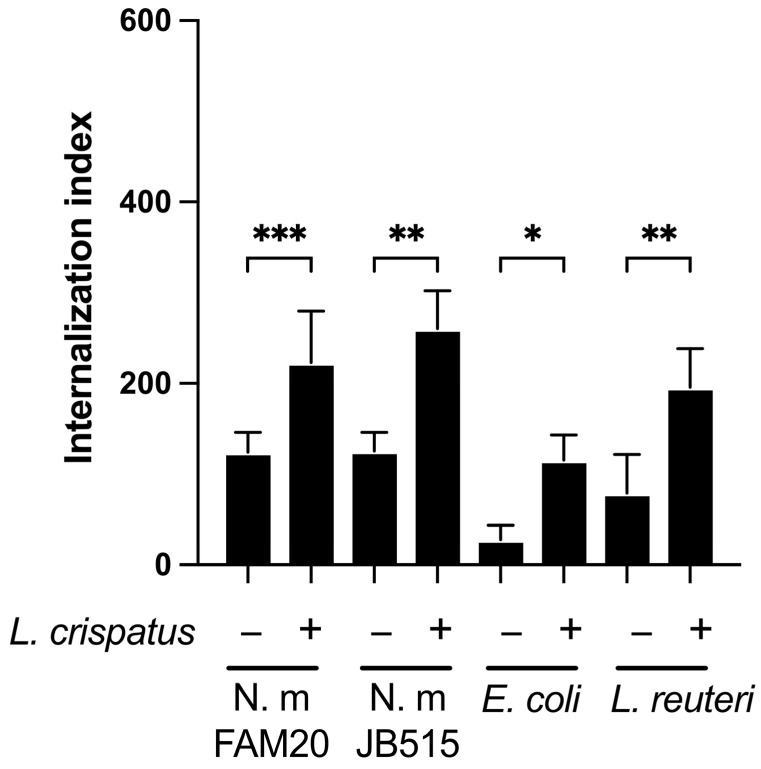
*L. crispatus* enhances the epithelial uptake of both pathogenic and commensal bacteria. Invasion of different bacterial species into FaDu cells measured by flow cytometry. CFSE-stained bacteria were added to cells pre-incubated for 1 h with *L. crispatus* (+) or with medium alone (−). Extracellular bacteria were quenched by adding 0.2% Trypan blue before reading. Cells were gated as intact single cells, and 10,000 events were counted. Values are shown as the internalization index where the percentage of cells with a signal is multiplied by the mean fluorescent intensity MFI. *N. meningitidis *(N. m) strains JB515 and FAM20, *E. coli*, *L. reuteri* incubated with or without *L. crispatus*. Data represent the mean ± SD of three independent experiments in duplicate. *** *p* < 0.005; ** *p* < 0.01; * *p* < 0.05.

Building on the observation that *L. crispatus* increased the uptake of fixed meningococci, we next investigated its effect on the invasion of other bacterial species into epithelial cells. Pharyngeal epithelial FaDu cells were pre-incubated with *L. crispatus* for 1 h followed by a 6 h co-incubation with either *N. meningitidis* JB515, *E. coli*, or *L. reuteri*. Co-incubation with *L. crispatus* increased the invasion of the *N. meningitidis* strain JB515 (serogroup W-135), mirroring the effect observed with strain FAM20 (Figure 3). Additionally, the internalization of *E. coli* O111:B4 and the non-pathogenic *L. reuteri* was also enhanced in the presence of *L. crispatus* (Figure 3). These findings collectively indicate that *L. crispatus* facilitates the entry of both pathogenic and non-pathogenic bacteria into epithelial cells.

### 3.4. L. crispatus Can Internalize Itself in Both Immortalized and Primary Epithelial Cells

**Figure 4:**
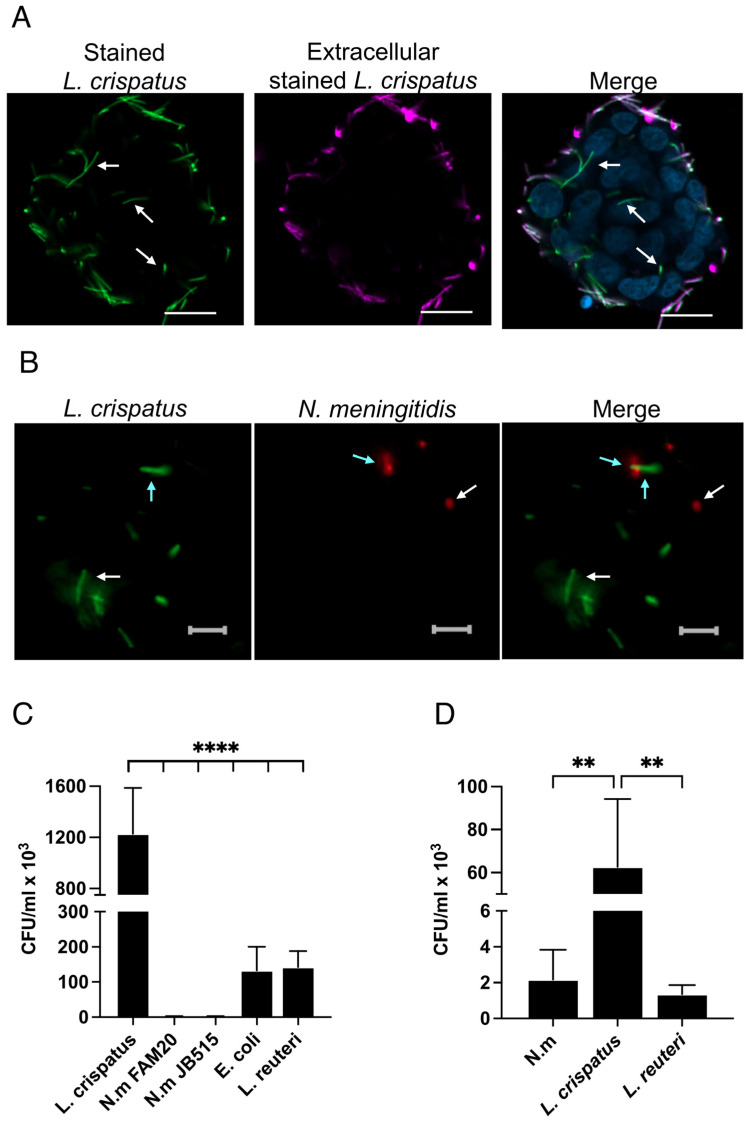
*L. crispatus* can internalize itself in both immortalized and primary cells. (**A**,**B**) Internalized bacteria were analyzed by confocal and wide-field fluorescence microscopy. Before incubation with cells, *L. crispatus* was stained with CellTrace^TM^ CFSE (green), *N. meningididis* with CellTrace^TM^ Far Red, and both bacteria were stained with Pierce^TM^ sulfo-NHS-LC-biotin. Next, FaDu cells were pre-incubated with *L. crispatus* for 1 h, infected with *N. meningitidis* for 6 h, and stained with Streptavidin-Alexa 568 to exclude non-intracellular bacteria, and cells were stained with Hoechst 33342. Cells were fixed with 4% PFA. (**A**) Mono-culture of stained *L. crispatus*. Images were obtained using a Zeiss LSM 800 with Airy Scan (Carl Zeiss AB, Oberkochen, Germany. White arrows indicate internalized *L. crispatus.* Scale bar 20 µm. (**B**) Co-culture with internalized *L. crispatus* and *N. meningitidis*. Images were obtained using a Zeiss Widefield Axio Observer 7 microscope (Carl Zeiss AB, Oberkochen, Germany. Light blue arrows indicate co-localized bacteria, and white indicates internalized bacteria that were not co-localized. Scale bar 5 µm. (**C**,**D**) Quantification of bacteria inside (**C**) FaDu cells or (**D**) Human Nasal Epithelial Cells (HNEpCs) using the gentamicin assay. Cells were incubated with each bacterial species individually for 6 h, treated with gentamicin for 1 h, and spread on plates to determine CFU/mL. Strains used: *N. meningitidis* (N. m) FAM20 and JB515, *E. coli, L. crispatus*, or *L. reuteri*. Data represent the mean ± SD of three independent experiments in duplicate. **** *p* < 0.001; ** *p* < 0.01.

Our data suggest that *L. crispatus* can increase the uptake of both *N. meningitidis* and other bacterial species through a process requiring direct physical contact with host cells. Recent studies have reported that other lactobacilli, i.e., *L. rhamnosus* and *L. paracasei,* can be internalized by the host cells [21]. To investigate whether *L. crispatus* similarly enters host cells and co-localizes with *N. meningitidis*, we used fluorescence microscopy. We observed *L. crispatus* inside host cells (Figure 4A) and sometimes found them co-localized with *N. meningitidis* (Figure 4B). To quantify intracellular bacteria, FaDu cells were incubated individually with different bacterial strains for 6 h, followed by enumeration using a gentamicin assay. The number of internalized *L. crispatus* was at least 10-fold higher compared to *L. reuteri* and *E. coli* (Figure 4C). As expected, *N. meningitidis* entered the cells at very low levels.

Given that *L. crispatus* primarily resides in the vaginal mucosa and that lactobacilli are known to decrease pH by secretion of lactic acid, we examined whether pH might influence its internalization. Incubations at pH of 5.4, 6.4, and 7.4 revealed no significant differences in *L. crispatus* uptake (Figure S3), suggesting that that pH changes might not affect internalization.

To verify the finding in a primary cell line, we used the primary nasal epithelial cell line HNEpC. Gentamicin assays demonstrated a high level of entry of *L. crispatus* compared to *N. meningitidis* and *L. reuteri* (Figure 4D). These data indicate that the internalization of *L. crispatus* far exceeds that of the other tested bacteria in both the immortalized FaDu pharyngeal cell line and in primary nasal epithelial cells, highlighting its unique capability to become internalized in epithelial cells.

### 3.5. Internalization of L. crispatus via the Caveolin-Mediated Endocytic Pathway

**Figure 5:**
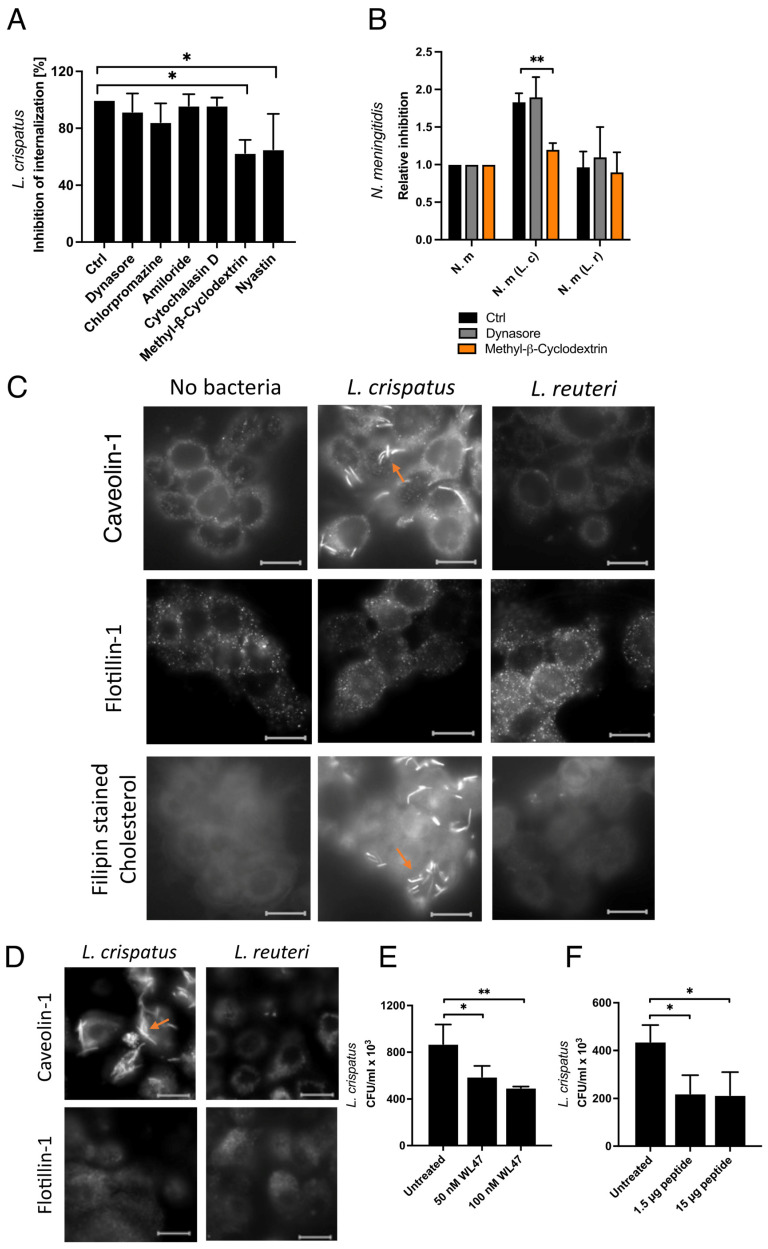
Internalization of *L. crispatus* via caveolin-mediated endocytosis. (**A**) Internalization of *L. crispatus* into FaDu cells. Cells were pre-incubated for 45 min with different chemical inhibitors of the endocytic pathways prior to incubation of *L. crispatus* for 6 h. After treatment with gentamicin to kill extracellular bacteria, bacteria were spread for viable count. (**B**) Internalization of *N. meningitidis* into FaDu cells. Host cells were pre-incubated with chemical inhibitors for 45 min and then with either *L. crispatus* or *L. reuteri* for 1 h. After infection with *N. meningitidis* for 6 h, cells were treated with gentamicin, and bacteria were spread for a viable count. Data represent the mean ± SD of three independent experiments in duplicate. * *p* < 0.05. (**C**) Fluorescence microscopy using polyclonal antibody against caveolin-1 or flotillin-1 with secondary antibody conjugated with Alexa-488 or Alexa-594, respectively. Cholesterol was detected with the cholesterol-binding chemical filipin. FaDu cells were inoculated with no bacteria, *L crispatus,* or *L. reuteri* for 6 h. (**D**) Primary Human Nasal Epithelial Cells (HNEpCs) inoculated with *L crispatus* or *L. reuteri*. Orange arrows highlight some of the areas where lipid raft-associated molecules have accumulated around bacteria. Scale bar 20 µm. Caveolin-1 inhibitors WL47 (**E**) and a blocking peptide (GTX89541-PEP) (**F**) were added to cells 30 min before incubation with *L. crispatus*. After incubation for 6 h, a gentamicin assay was performed and bacteria were spread for viable count. ** *p* < 0.01; * *p* < 0.05; unmarked bars are considered nonsignificant.

Data showed that *L. crispatus* became internalized into epithelial cells at high numbers. To identify the endocytic pathway responsible for this uptake, we used inhibitors of phagocytosis/micropinocytosis, clathrin/receptor-mediated endocytosis, and caveolin/lipid raft-mediated endocytosis. Neither dynasore nor chlorpromazine, which inhibits clathrin-mediated endocytosis, reduced the internalization of *L. crispatus*. Similarly, amiloride, an inhibitor of micropinocytosis, and cytochalasin D, which inhibits phagocytosis by preventing actin polymerization, failed to block the uptake of *L. crispatus*. These results suggest that *L. crispatus* does not utilize phagocytosis/micropinocytosis or clathrin/receptor-mediated pathways for internalization (Figure 5A). We then investigated the remaining pathway, i.e., caveolin/lipid raft-mediated endocytosis. To assess this pathway, we used methyl-β-cyclodextrin, which depletes cholesterol from the plasma membrane, thereby inhibiting the invagination of the plasma membrane. Treatment with methyl-β-cyclodextrin significantly inhibited the internalization of *L. crispatus*. Similarly, nystatin, which decomposes cholesterol and inhibits caveolae/lipid-mediated endocytosis, also reduced the uptake of *L. crispatus* (Figure 5A). These results indicate that *L. crispatus* is internalized via a caveolae/lipid raft-dependent mechanism.

To further investigate the role of this pathway in facilitating meningococcal uptake during co-incubation, we treated cells with two inhibitors: methyl-β-cyclodextrin, which inhibits *L. crispatus* internalization, and dynasore, which does not. Entry of *N. meningitidis* alone was unaffected by either inhibitor. However, during co-incubation with *L. crispatus*, the inhibition of caveolae/lipid raft-mediated endocytosis by methyl-β-cyclodextrin blocked *N. meningitidis* internalization (Figure 5B). We used *L. reuteri*, unable to enhance meningococcal invasion, as a negative control. These findings suggest that caveolae/lipid raft-mediated endocytosis is involved in a *L. crispatus*-mediated increase in *N. meningitidis* internalization.

### 3.6. Caveolin and Cholesterol but Not Flotillin Were Recruited Around L. crispatus

To confirm the involvement of caveolae-mediated endocytosis in *L. crispatus* internalization, we examined whether caveolin-1 or flotillin-1 was associated with *L. crispatus*. Caveolin-1 plays a central role in caveolae-formation and stability, while flotillin-1 is associated with non-caveolar microdomains/lipid rafts. Using fluorescently labeled antibodies against caveolin-1 and flotillin-1, as well as filipin, a fluorescent dye that binds to cholesterol, we observed the recruitment of caveolin-1 and cholesterol around *L. crispatus* in FaDu pharyngeal cells. In contrast, flotillin-1 was not recruited to these sites (Figure 5C). To verify that this also occurred in primary cells, we analyzed caveolin-1 recruitment in primary human nasal epithelial (HNEpCs) cells. Similar to the results in FaDu cells, caveolin-1 accumulated around *L. crispatus* in HNEpC cells (Figure 5D). Importantly, this accumulation of caveolin-1 and cholesterol was not observed around the non-internalized control bacterium, *L. reuteri*.

To further prove the involvement of caveolin-1 in *L. crispatus* internalization, we used a selective caveolin-1 oligomer disruptor WL47 as well as a blocking peptide that blocks the possibility of caveolin-1 interacting with targets. Both these inhibitors strongly reduced the internalization of *L. crispatus* (Figure 5E,F). In summary, the finding that caveolin-1 and cholesterol, key molecules required for caveolae-mediated endocytosis, accumulated around sites where *L. crispatus* adheres and the finding that inhibition of caveolin-1 reduced uptake of *L. crispatus* support a role of caveolin-1 dependent pathways in *L. crispatus* internalization.

### 3.7. L. crispatus Survives Inside Cells, Whereas N. meningitidis Is Killed over Time

**Figure 6:**
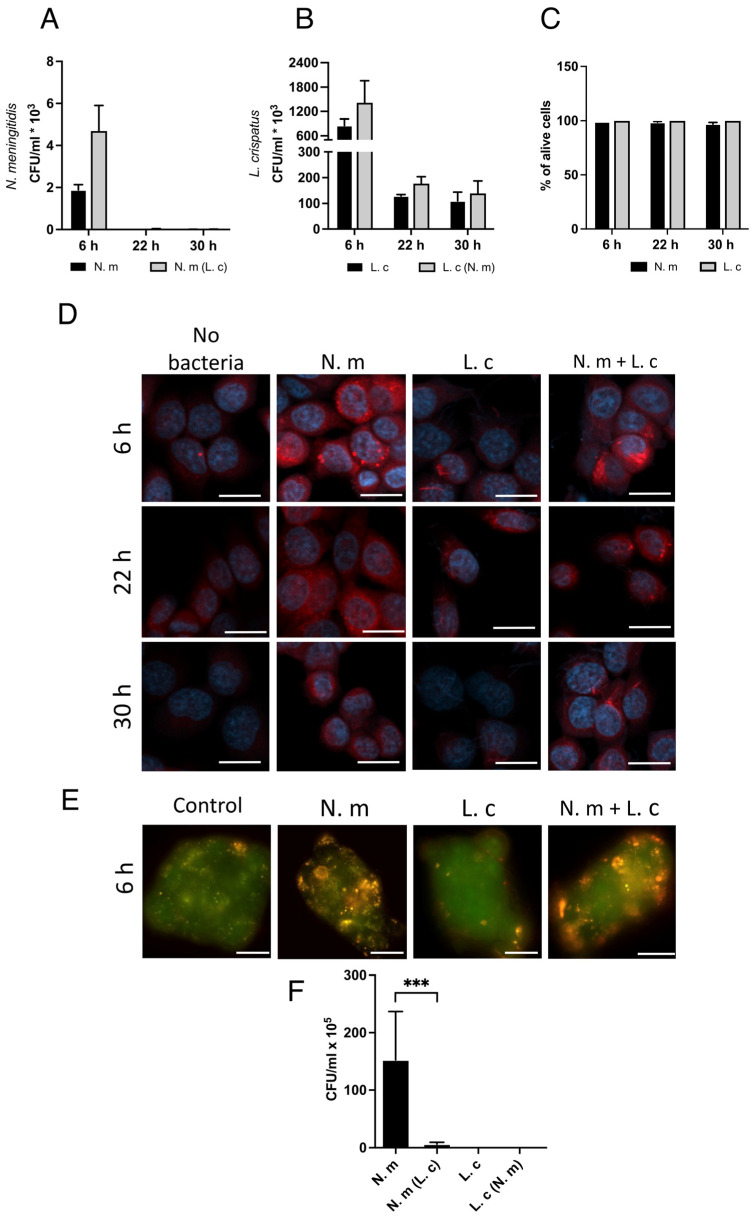
Co-incubation with *L. crispatus* prevents *N. meningitidis* from exiting epithelial cells. Cells were pre-incubated for 1 h with or without *L crispatus* (L. c) before infection with *N*. *meningitidis* (N. m) for 6 h, 22 h, or 30 h. After treatment with gentamicin for 1 h to kill extracellular bacteria, bacteria were plated on GC-plates (*N. meningitidis*) or Rogosa plates (lactobacilli) for viable counts. (**A**) Internalized *N. meningitidis* with or without *L. crispatus*. (**B**) Internalized *L. crispatus* with or without *N. meningitidis*. (**C**) Live/dead stain using flow cytometry to assess survival of FaDu cells. Data represent the mean of three independent experiments in triplicate. (**D**) Staining of infected cells for acidic vacuoles. Cells were pre-incubated for 1 h with or without *L crispatus* (L. c) before infection with *N*. *meningitidis* (N. m) for 6 h, 22 h, or 30 h. Post-infection, the cells were stained with LysoTracker™ Red DND-99 (Invitrogen, Carlsbad, CA, USA), fixed, and imaged with Zeiss LSM 800 with an Airy scan (Carl Zeiss AB, Oberkochen, Germany) at 20× magnification. Scale bars indicate 20 µm. (**E**) Staining of infected cells for acidic vacuoles using acridine orange stain. Cells were pre-incubated with *L. crispatus* for 1 h before infection with *N. meningitidis* for 6 h. Cells were imaged using a Zeiss Widefield Axio Observer 7 microscope at 63x magnification. Scale bars indicate 20 µm. (**F**) Bacteria released from a cell layer maintained on 5-µm transwell membranes. Cells were pre-incubated for 1 h with or without *L crispatus* (L. c) before infection with *N*. *meningitidis* (N. m) for 6 h. The number of bacteria in the lower compartment was plated for viable count using GC-plates for *N. meningitidis* and Rogosa plates for *L. crispatus*. Data represent the mean ± SD of three independent experiments in triplicate. *** *p* < 0.005; unmarked bars are considered nonsignificant.

To study the survival of internalized bacteria, we enumerated intracellular bacteria not only at 6 h but also at 22 h and 30 h of co-incubation. After treatment with gentamicin for 1 h, the intracellular bacteria were plated for a viable count. *N. meningitidis* did not survive inside cells at the 22 h time point, regardless of co-incubation with *L. crispatus* (Figure 6A). On the other hand, the lactobacilli survived inside cells during the tested time span, although the amount initially decreased at 22 h but then remained stable up to the last tested time point at 30 h. (Figure 6B). None of the bacterial strains affected cell viability according to a live/dead assay (Figure 6C).

To study the possible differences in host cell response to bacterial entry, we stained infected cells with LysoTracker Red DND-99, which detects lysosomal abundance and acidity. At the 6 h time point, cells containing *N. meningitidis* showed a striking increase in LysoTracker staining (Figure 6D), while the staining had decreased intensity at 22 h and back to control levels at 30 h of incubation. The increase in LysoTracker staining may reflect an increase in lysosome numbers or increases in their size. Epithelial cells with only lactobacilli remained negative at all three time points, similar to the control with cells alone (Figure 6D).

In separate experiments, we used the weak base acridine orange, which enters cells and accumulates in the acidic environment of lysosomes. Cells infected with *N. meningitidis* showed a strong signal with acridine orange, while cells incubated with *L. crispatus* showed no staining, similar to the negative control (Figure 6E). All in all, both lysosome tracker and acridine orange staining indicated that invasion of *N. meningitidis* increased acidic vacuoles, whereas internalization of lactobacilli did not, which is in line with meningococcal elimination and survival of lactobacilli.

### 3.8. L. crispatus Prevents N. meningitidis from Exiting Epithelial Cells

Next, we investigated how the presence of *L. crispatus* might affect the release of bacteria from cells. To do this, we added bacteria to a layer of epithelial cells grown on transwell inserts with pore size of 5 µm and then collected bacteria in the lower compartment after 6 h. *N. meningitidis* alone transcytozed in high numbers, whereas the exit of *N. meningitidis* from the cells was strongly inhibited by co-incubation with *L. crispatus* (Figure 6F). *L. crispatus* did not pass through the cell layer alone or in wells together with *N. meningitidis* (Figure 6F). All bacteria were tested and confirmed to be able to cross the transwell membrane without cells. Measurement of *trans-epithelial* electrical *resistance* (TEER) and the passage of luciferase yellow confirmed a tight layer of cells (Figure S4). In control experiments, *L. crispatus* entered the cells and increased the uptake of *N. meningitidis* maintained on transwells (Figure S4), similar to that previously shown for cells maintained in a microtiter plate. Taken together, these data suggest that co-incubation with *L. crispatus* prevents the transcellular passage of *N. meningitidis*.

## 4. Discussion

Normal flora is an important first line of defense against invading pathogens. Lactobacilli are common inhabitants at mucosal membranes of the oropharyngeal and gastric tract and are known to protect against colonization of many pathogens. However, the molecular mechanisms of how *Lactobacillus* can interfere with pathogens remain largely unknown. In this study, we showed that *L. crispatus* efficiently entered epithelial cells via caveolin-mediated endocytosis, and blocking this pathway inhibited the entry of *L. crispatus.* The presence of lactobacilli also inhibited the exit of *N. meningitidis* from the cells, which led to the destruction of the pathogen via acidic vacuoles, while lactobacilli survived.

Four *Lactobacillus* strains were chosen for initial screening experiments to investigate the interaction with *N. meningitidis*. Two oral and two vaginal isolates with described anti-pathogenic effects [26,38,39] were relevant due to their association with the nasopharyngeal mucosa. None of the strains tested had an effect on *N. meningitidis* adherence to host cells, while *L. crispatus* affected pathogen uptake into epithelial cells. For further experiments, *L. reuteri* was chosen as a negative control since it did not influence internalization levels of itself or *N. meningitidis* into host cells. Furthermore, it did not significantly co-localize with caveolin. The *Lactobacillus* genus is diverse, and even among *L. crispatus* species, there is a range of functions. In a study investigating the microbiota from vaginal dysbiosis compared to healthy mucosa, *L. crispatus* was found to consume glycogen in the isolates from healthy individuals but not in the patients with dysbiosis [40]. The strain used in this study was obtained from a healthy human donor. A more in-depth study comparing different lactobacilli from distinct niches would be an important future direction regarding caveolin-mediated endocytosis.

The finding that *L. crispatus* independently internalizes and enhances the uptake of *N. meningitidis* as well as other pathogens and commensal species in both the immortalized cell line FaDu and primary nasal epithelial cells (HNEpCs) is intriguing. The internalization of commensals into host epithelial cells is a new but important concept. Ramirez-Sánchez et al. [21] found that *Lacticaseibacillus paracasei* and *Lacticaseibacillus rhamnosus* were internalized by intestinal epithelial cells. Here, we demonstrated that the commensal *L. crispatus* not only internalizes into pharyngeal epithelial cells but that this spontaneous internalization affects pathogenic uptake. The mechanism of entry by *L. crispatus* differed from the previous finding of Ramirez-Sánchez et al., which involved actin filament arrangements. In this work, we found that the entry of *L. crispatus* occurred via caveolin-mediated endocytosis and involved the recruitment of caveolin-1 and cholesterol to the lactobacilli adherence site.

The ability of *N. meningitidis* to invade host cells is fundamental to its pathogenicity, allowing it to evade host immune defenses, cross cellular barriers, and disseminate to cause sepsis and meningitis [41,42]. *N. meningitidis* colonizes epithelial surfaces and can invade the host by itself but is considered an epicellular bacterium [43]. Upon close contact between bacteria and the cell surface, CrgA downregulates the capsule, enabling Opa-CEACAM binding. This triggers endocytosis and transmigration, where, again, *N. meningitidis* needs capsule upregulation to survive [44,45,46]. In this work, the uptake of *N. meningitidis* increased in co-culture with *L. crispatus* but not with other tested lactobacilli, and this increase in internalization was not dependent on the CEACAMs of the host. Although we tested several invasion-associated factors, such as CEACAM and virulence gene expression, we cannot exclude that other invasion-related factors, either on the host cells or bacterial virulence molecules, could play a role in the increased meningococcal invasion observed in the presence of *L. crispatus*.

We found that *L. crispatus* needed to be in contact with host cells to increase meningococcal invasion. The nasopharyngeal epithelium is embedded in 0- to 12-μm-thick two-layer surface liquid, mucosal MUC5A and MUC5B-rich mucus, with a low-viscosity layer close to the epithelium, and a denser mucus facing the lumen [47,48,49]. This might also affect the ability of lactobacilli to come in contact with host cells. Furthermore, the nasopharyngeal niche has a higher pH in the range of 6.1–7.9 compared to the vaginal niche [50]. Therefore, it was essential to investigate whether pH might affect how the lactobacilli behave. We tested a lower pH of the cell medium to determine whether the pH affected *L. crispatus*-induced meningococcal uptake, but the result showed no difference.

Not only *N. meningitidis* but also the other tested bacterial species, *E.coli* and *L. reuteri,* showed increased internalization in FaDu cells when co-incubated with *L. crispatus*. We cannot be sure that this *L. crispatus*-induced trafficking of bacteria is a host-beneficiary effect, as other pathogens may take advantage of the increased internalization. The respiratory epithelium can also be colonized by pathogens such as *Staphylococcus aureus, Streptococcus pneumoniae, Haemophilus influenzae*, and *Mycobacterium tuberculosis,* which all have a strategy to resist intracellular killing [48,51]. It is possible that these pathogens may use lactobacilli in a similar way, and it would be interesting to examine this in the future. It may also be that the presence of lactobacilli may facilitate uptake but not prevent pathogen destruction and/or release from host cells.

It has been shown that internalized *N. meningitidis* exits the cell in an induced membrane-dependent exit without host-cell lysis [43,52,53]. In this work, we used TEER and staining marker lucifer yellow to verify that cell layers stayed intact during experiments. Interestingly, *L. crispatus* alone did not pass the cell layer to reach the lower chamber below the transwell membrane, and importantly, the commensal strain prevented transcytosis of meningococci. This way to limiting the spread of the pathogen into adjacent cells could contribute to faster clearance of the infecting agent and prevent dissemination.

We observed a rapid killing of *N. meningitidis* inside cells and an increased level of acidic vacuoles. Dzidic et al. [9] have shown that *L. crispatus* can be found in the nasopharynx in children born vaginally and reside there until up to seven years of age. Intriguingly, it could be speculated that the presence or absence of *L. crispatus* might reduce the risk of meningococcal disease in children of all ages, potentially as early as from birth. The neonatal incident rate for invasive meningococcal disease is low [54]. However, clinical data indicate that meningococci can infect the neonates intrauterinely, intrapartum, or post-partum [54]. Here, vaginal lactobacilli, such as *L. crispatus*, could play a protective role pre-birth, since the anti-pathogenic effects of *L. crispatus* are well documented in the vaginal mucosa [55,56]. Additionally, *L. crispatus* is a well-known, safe, and widely used probiotic in clinical trials [57,58]. Future clinical studies of meningococcal risk groups would be of interest.

There are still exciting questions to be addressed in future research. One such question is the mechanism by which *L. crispatus* influences the host to increase the uptake of itself and other bacteria. Once internalized, how does the cellular processing of the pathogen differ from when it actively invades versus *L. crispatus*-mediated uptake? The increase in acetic vacuoles and clearance of the pathogen were equally effective in both mono- and co-culture, but the exocytosis of the pathogen decreased when co-present together with the lactobacilli, why? Do lactobacilli impede the polarization described by *N. meningitidis* as detailed by Barillé et al. [8]? Our data did not show a significant difference in TEER measurement. However, we did not design the experiment specifically to look into this, and it could be an interesting line of investigation. Lastly, although we demonstrated pathogen clearance in epithelial cells, expanding the picture would be needed, both with subsequent immune response investigation in vitro experiments, followed by in vivo experiments and animal studies. These questions, while important, are beyond the scope of this study.

## 5. Conclusions

In conclusion, the data in this work demonstrate that *L. crispatus* can be internalized into the host epithelium without increasing acidic vacuoles and survive for up to 30 h without harming the host. The recruitment of lipid raft-associated molecules caveolin and cholesterol at the adherence site of *L. crispatus* is linked to the observed internalization. Furthermore, in co-culture with pathogenic *N. meningitidis*, the pathogen uptake increased. Nevertheless, transcytosis from the cell layer was reduced and increased acidic vacuole levels led to clearance of the pathogen but did not cause clearance of lactobacilli. This study demonstrates that lactobacilli can be internalized into the host while increasing the uptake of *N. meningitidis* for destruction, which could have clinical relevance in clearing bacterial pathogens.

## Data Availability

The datasets used and/or analyzed during the current study are available from the corresponding author upon reasonable request.

## Supplementary Materials

The following supporting information can be downloaded at: https://www.mdpi.com/article/10.3390/microorganisms13030479/s1. Figure S1. Uncropped versions of images used in the main experiments. Figure S2. Lactobacilli did not affect viability of *N. meningitidis.* Figure S3. Acidification of the media did not affect internalization of *L. crispatus.* Figure S4. Confirmation of cell layer integrity and internalization assay of bacteria on transwell membranes. Table S1. Bacteria used in this study. Table S2. Raw data of the main experiments containing bacteria counting. Table S3. Primers used in this study [59,60,61].
