# Supplementary material for: Internalization of Lactobacillus crispatus Through Caveolin-1-Mediated Endocytosis Boosts Cellular Uptake but Blocks the Transcellular Passage of Neisseria meningitidis
Source: Microorganisms. 2025 Feb 21;13(3):479. doi: 10.3390/microorganisms13030479 (PMC11945323; doi:10.3390/microorganisms13030479)
Supplement: Supplementary file 1 [file microorganisms-13-00479-s001.zip › microorganisms-3434455-supplementary.pdf]

Supplementary Material

Internalization of *Lactobacillus crispatus* Through Caveolin-1-Mediated Endocytosis Boosts Cellular Uptake but Blocks the Transcellular Passage of *Neisseria meningitidis*

Kenny Lidberg,[1] Sarah Pilheden,[1] Mikel Relloso Ortiz de Uriarte,[1] and Ann-Beth Jonsson[1*]

[*] Corresponding author: Ann-Beth Jonsson, ann-beth.jonsson@su.se

FIGURE S1

A

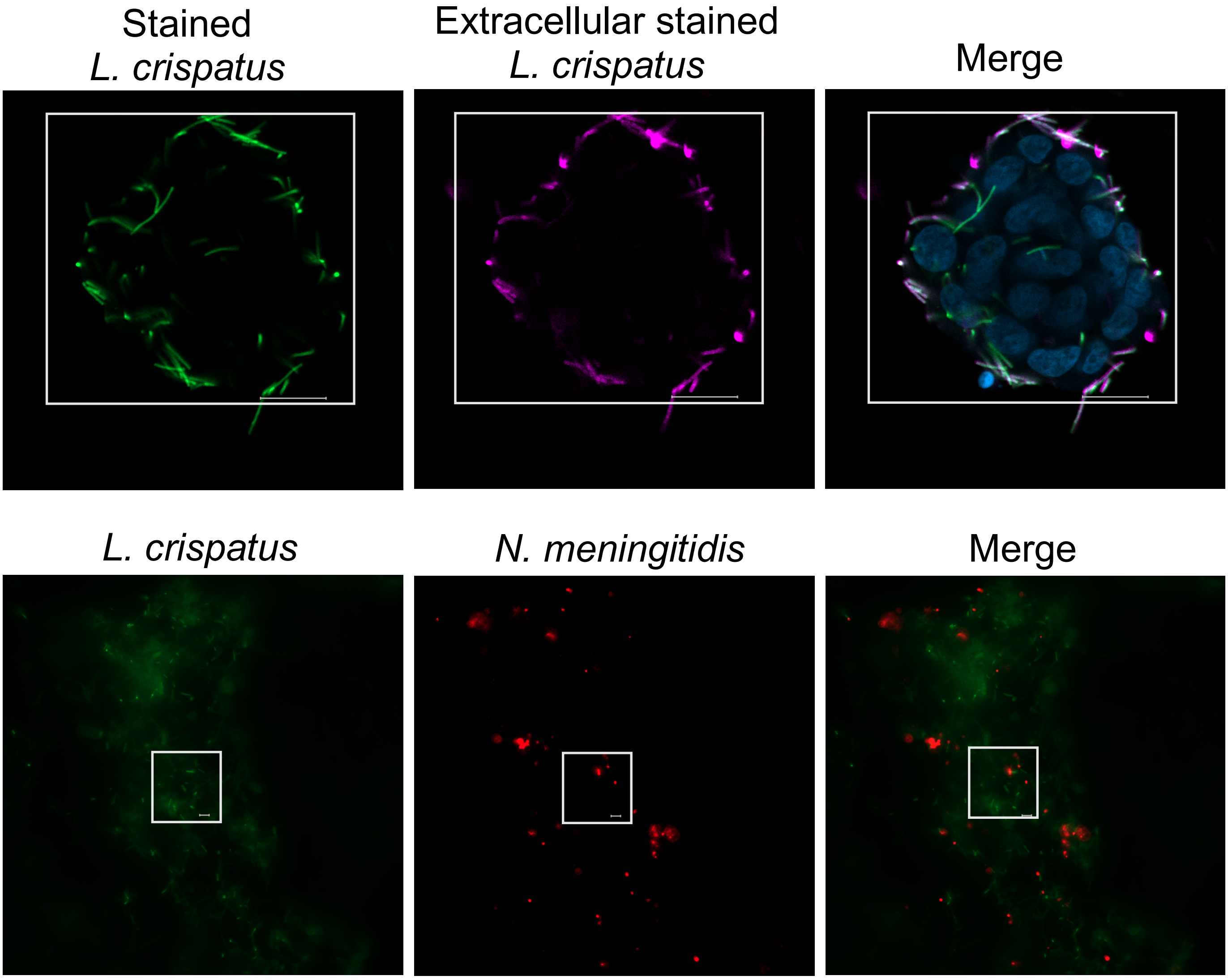
Stained
L. crispatus
Extracellular stained
L. crispatus
Merge
L. crispatus
N. meningitidis
Merge

B

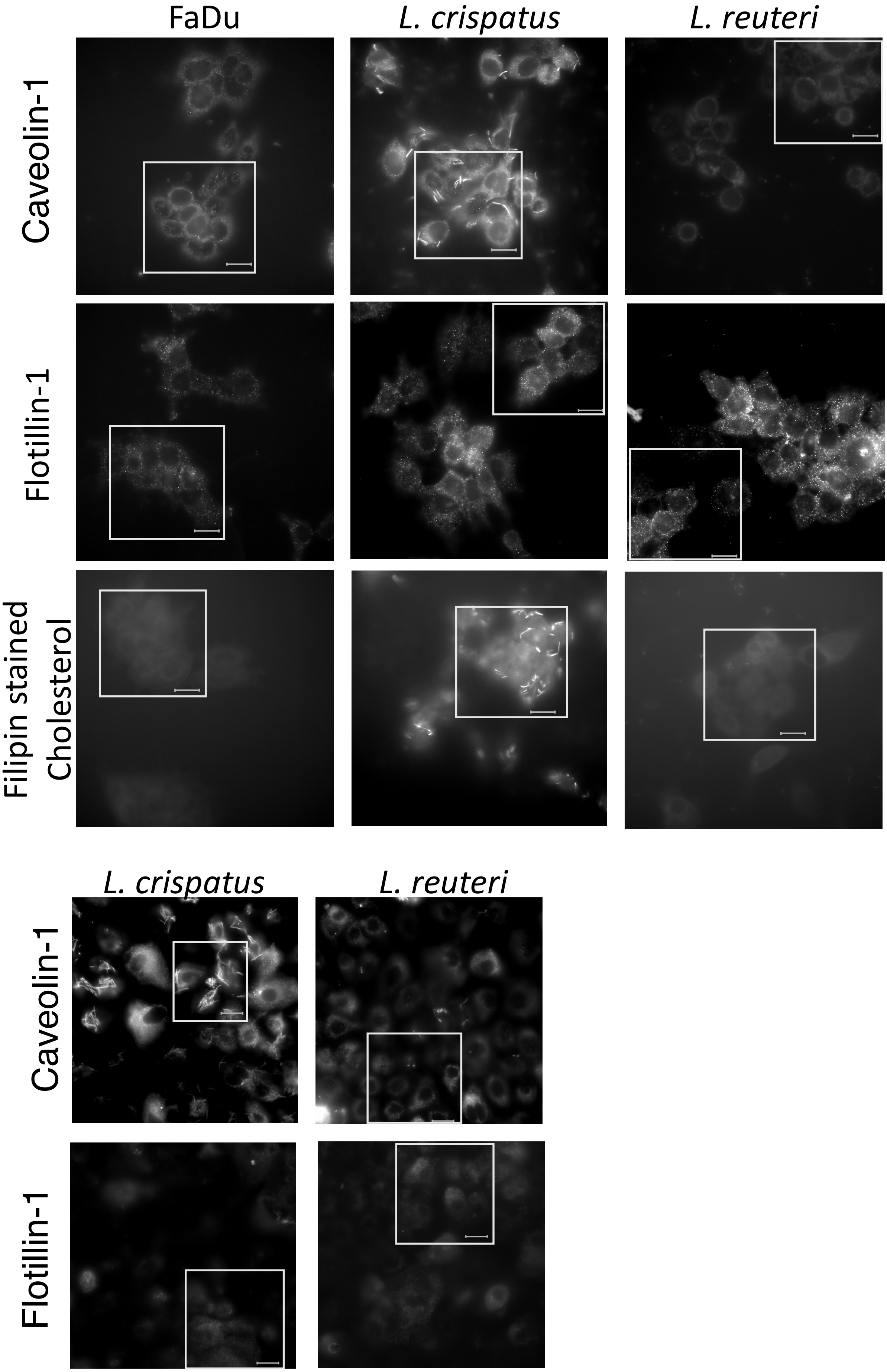
FaDu
L. crispatus
L. reuteri
Caveolin-1
Flotillin-1
Filipin stained Cholesterol
L. crispatus
L. reuteri
Caveolin-1
Flotillin-1

C

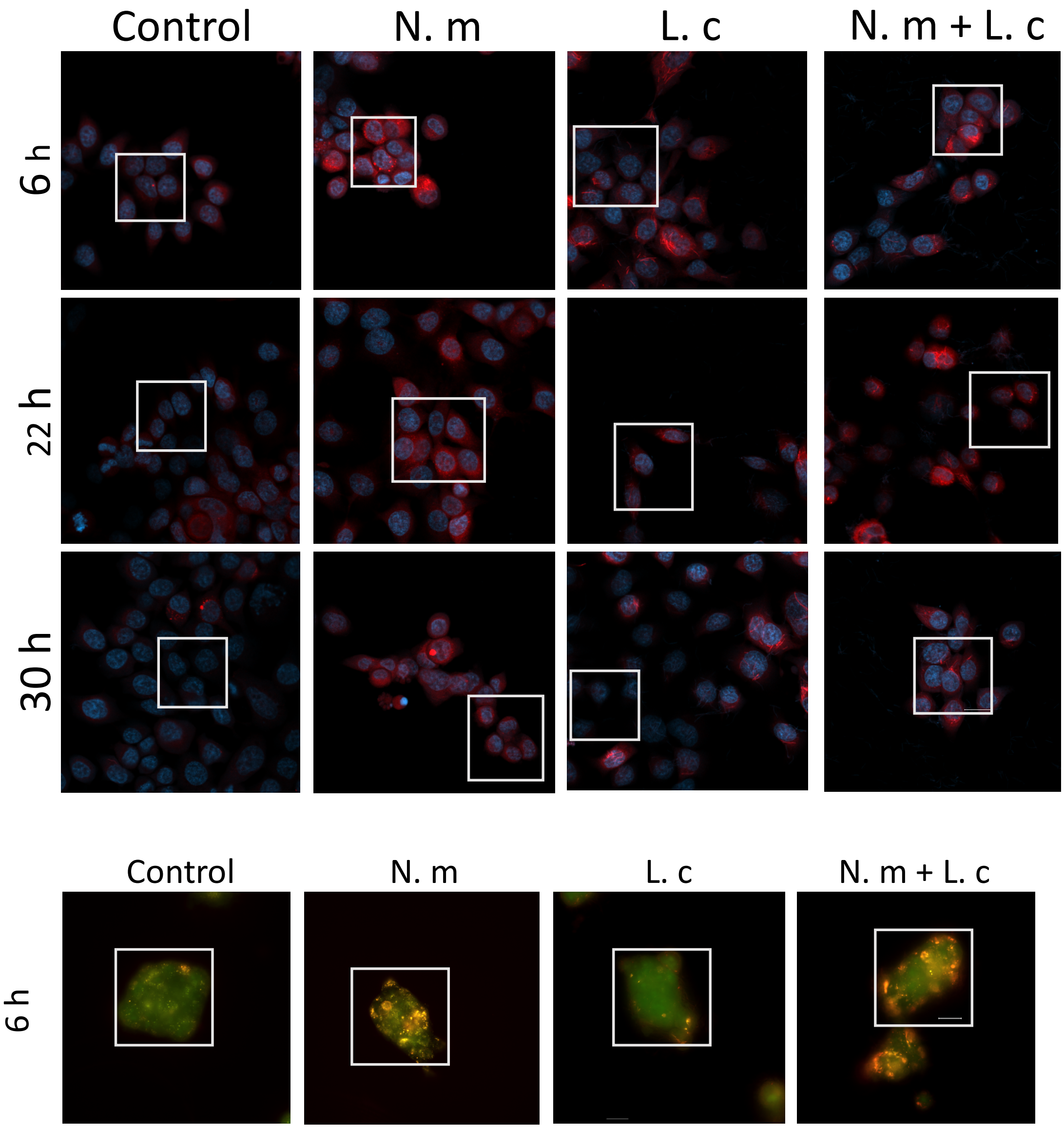

Figure S1. Uncropped versions of images used in the main experiments
(A) Uncropped images from Figures 4A and 4B. (B) Uncropped images from Figures 5C and 5D. (C) Uncropped images from Figures 6D and 6E. The white box indicates where the images were cropped.

FIGURE S2

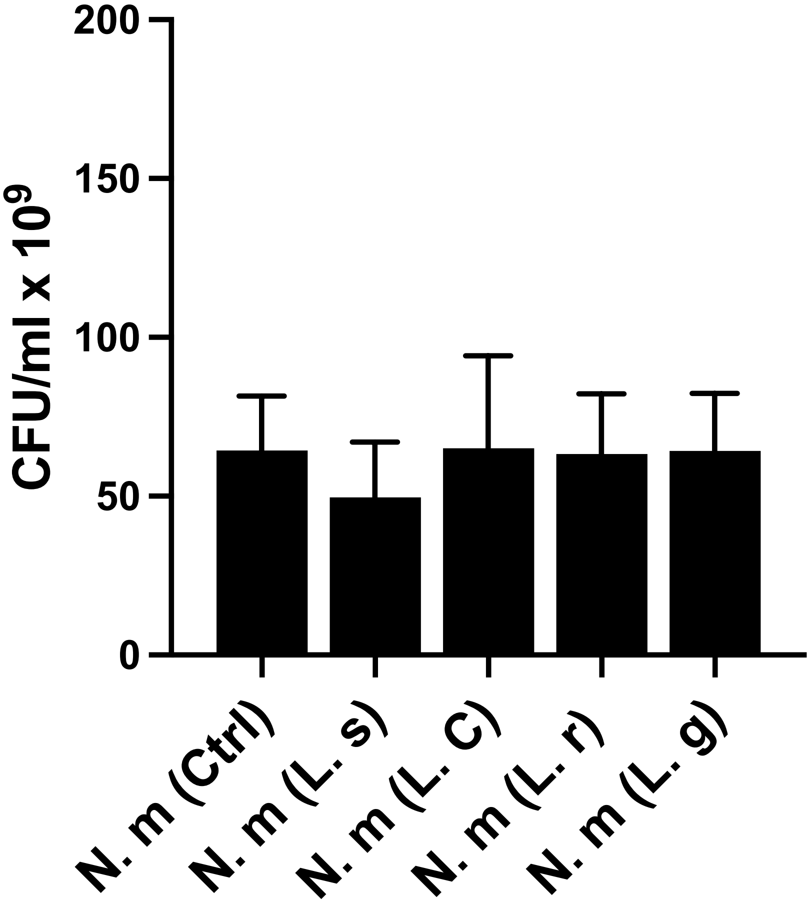

Figure S2. Lactobacilli did not affect viability of *N. meningitidis*

Viability of *N. meningitidis* (N. m) in the presence and absence of *L. salivarius* (L. s), *L crispatus* (L. c), *L. reuteri* (L. r), *L. gasseri* (L. g) in the medium used in this study (DMEM with 1% FBS and 1 mM lactate). Bacteria were incubated for 6 h, serially diluted, and plated for viable count. Data represent the mean of three independent experiments in triplicate. Unmarked bars are considered nonsignificant.

FIGURE S3

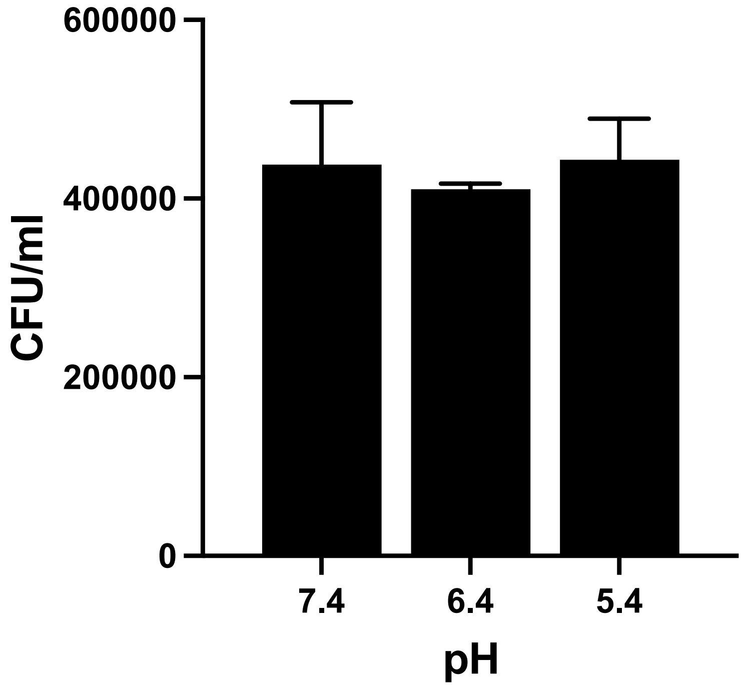

Figure S3. Acidification of the media did not affect internalization of *L. crispatus*

Internalization of *L. crispatus* using gentamicin invasion assay. *L. crispatus* was incubated with FaDu cells for 6 h, treated with gentamicin for 1 h to kill extracellular bacteria, serially diluted and spread on plates to determine CFU/ml. The medium was originally at pH 7.4 and was acidified using lactic acid to pH 6.4 or 5.4. Data represent the mean ± SD of two independent experiments in triplicates. Unmarked bars are considered nonsignificant.

FIGURE S4

A

	Mean Values at 0 h		Mean values at 6 h		
	TEER	Unit area resisance	TEER	Unit area resisance	% LY
N. m (Ctrl)	195,5	19,9	200,4	22,0	12,5
N. m (L. c)	193,5	19,3	197,1	21,1	14,4
L. c	194,7	19,9	197,5	21,3	13,6
No bacteria (Ctrl)	192,3	19,3	184,3	17,8	15,0
No cells (Ctrl)	129,2	0,0	126,8	0,0	74,4

B C

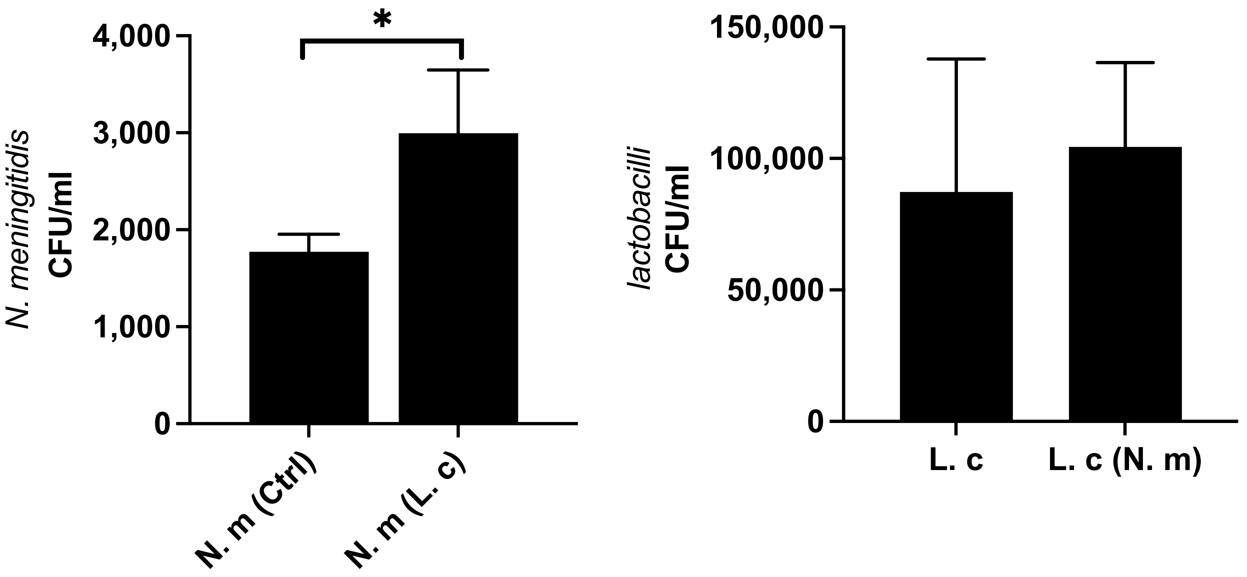

Figure S4. Confirmation of cell layer integrity and internalization assay of bacteria on transwell membranes

(A) Measurement of trans epithelial electrical resistance (TEER) at 0 h and 6 h and luciferase yellow (%LY) at 6 h in transwell experiments. Cells were maintained on 5 μm transwell membranes and incubated with bacteria or control medium. Cells were pre-incubated for 1 h with *L crispatus* (L. c) or medium (Ctrl) before infection with *N. meningitidis* (N.m). Lactobacilli were also kept alone in wells. (B, C) Internalization of *N. meningitidis* and lactobacilli using gentamicin assay on cells cultured on transwell inserts. Cells were preincubated with lactobacilli for 1 h and infected with *N. meningitidis* for 6 h. After treatment with gentamicin for 1 h to kill extracellular bacteria, cells were plated for viable counts. (B) *N. meningitidis* was plated on GC-plates. (C) Lactobacilli were plated on Rogosa-plates. Data represent the mean ± SD of three independent experiments in triplicates. *$p<0.05$; unmarked bars are considered nonsignificant.

Table S1. Bacterial strains used in this study

Species	Strain	Origin of isolation
Neisseria meningitidis	FAM20 and JB515	Patient with septicemia
Ligilactobacillus salivarius	LM9477	Human saliva from a healthy donor
Lactobacillus crispatus	MV24-1A	Human vagina from a healthy donor
Lactobacillus gasseri	MV1-1A	Human vagina from a healthy donor
Limisilactobacillus reuteri	ATCC PTA5289	Human saliva from a healthy donor
Escherichia coli	O111:B4	Human newborn stool

Table S3. Primers used in this study

Primers	Sequence (5'-3')	Reference
gdh_fwd	TCG CCA TTA AAG CCG AAA TC	59
gdh_rev	CTTG CCG GTA CGC AGG TAG A	59
siaD_fwd	CCT ACT ACC CAA TGT CTG TCA A	60
siaD_rev	GCT CTT CAA TTA AAG CGG TGT TC	60
crgA_fwd	CTCGTTGTGCCTTTCAGGTT	61
crgA_rev	TTAACTTTCCTTCAGCGATGTC	61
opaA1_fwd	AGG CAA GAC CTG AAG ACG GAA	This work
opaA1_rev	TGA AAT CGT AAA CGG CGG ACA A	This work
lpxA_fwd	ACC ACA CCA TCT TCG CCA AC	This work
lpxA_rev	CGA TGC GGC AGA ATT GGA AAA C	This work

Table S2. Raw data of main experiments containing bacterial counting

Figure 1A

Sample	Average CFU count	Average CFU/ml x 10^7	std	P- value	Significance
N. m (Ctrl), (n=3)	55	1.10	0.29		
N. m (L. s), (n=3)	56	1.11	0.39	>0.9999	ns
N. m (L. c), (n=3)	55	1.10	0.33	>0.9999	ns
N. m (L .r), (n=3)	58	1.17	0.23	0.8561	ns
N. m (L. g), (n=3)	60	1.20	0.33	0.2943	ns

Figure 1B

Sample	Average CFU count	Average CFU/ml	std	P- value	Significance
N. m (Ctrl), (n=3)	23	470	250		
N. m (L. s), (n=3)	20	401	154	>0.9999	ns
N. m (L. c), (n=3)	55	1096	411	0.0046	**
N. m (L .r), (n=3)	22	441	154	>0.9999	ns
N. m (L. g), (n=2)	19	384	90	>0.9999	ns

Figure 1D

Sample	Average CFU count	Average CFU/ml	std	P- value	Significance
N. m (Ctrl), (n=3)	18	723	40		
N. m (L.c), (n=2)	26	1025	92	0.0048	**
N. m (L.r), (n=3)	16	660	52	0.4874	ns

Figure 4B

Sample	Average CFU count	Average CFU/ml x 10^3	std	P- value	Significance
L. crispatus, (n=3)	307	1229	358		
N.m FAM20, (n=3)	52	2	1	<0.0001	****
N.m JB515, (n=3)	45	1.7	1.2	<0.0001	****
E. coli, (n=3)	33	133	68	<0.0001	****
L. reuteri, (n=3)	36	143	46	<0.0001	****

Figure 4C

Sample	Average CFU count	Average CFU/ml x 10^3	std	P- value	Significance
N.m, (n=4)	54	2.17	1.66	0.0044	**
L. crispatus, (n=4)	152	62.69	31.49		
L. reuteri, (n=3)	34	1.354	0.5137	0.0064	**

Figure 5A					
Sample	Average CFU count	Average reltive value [%]	std	P- value	Significance
Ctrl (n=4)	214	100	0		
Dynasore, (n=4)	200	92	12.7	>0.9999	ns
Chlorpromazine, (n=3)	198	84	13.2	0.8052	ns
Amiloride, (n=3)	225	96	7.9	>0.9999	ns
Cytochalasin D, (n=4)	206	96	5.6	0.8688	ns
Methyl-β-Cyclodextrin, (n=4)	154	63	9.2	0.009	**
Nyastin,(n=4)	147	65	24.9	0.0086	**

Figure 5B					
Sample	Average CFU count	Average reltive value	std	P- value	Significance
N.m Ctrl,(n=3)	93	1	0	N/A	N/A
N.m Dynasore, (n=3)	172	1	0	N/A	N/A
N.m Methyl-B-Cyclodextrin, (n=3)	87	1	0	N/A	N/A
N.m (L. c) Ctrl, (n=3)	97	1.9	0.05		
N.m (L. c) Dynasore, (n=3)	174	1.9	0.22	0.9564	ns
N.m (L. c) Methyl-B-Cyclodextrin,(n=3)	85	1.2	0.07	0.008	**
N.m (L. r) Ctrl, (n=3)	112	0.97	0.17		
N.m (L. r) Dynasore, (n=3)	133	1.1	0.33	0.6919	ns
N.m (L. r) Methyl-B-Cyclodextrin, (n=3)	99	0.9	0.22	0.2745	ns

Figure 5E					
Sample	Average CFU count	Average CFU/ml x 10^3	std	P- value	Significance
Untreated, (n=3)	223	870	167		
50 nM WL-47 (n=3)	152	591	91	0.0116	*
100 nM WL-47 (n=3)	125	496	11	0.0019	**

Figure 5F					
Sample	Average CFU count	Average CFU/ml x 10^3	std	P- value	Significance
Untreated (n=3)	109	437	69		
1.5 µg peptide, (n=3)	55	220	77	0.0346	*
15 µg peptide, (n=3)	54	214	96	0.0307	*

Figure 6A					
Sample	Average CFU count	Average CFU/ml x 10^3	std	P- value	Significance
N. m 6 h, (n=3)	95	1.9	0.20	N/A	N/A
N. m (L. c) 6 h, (n=2)	425	4.7	0.85	N/A	N/A
N. m 22 h, (n=3)	0	0.0	0.00	N/A	N/A
N. m (L. c) 22 h, (n=3)	1	0.0	0.02	N/A	N/A
N. m 30 h, (n=3)	0	0.0	0.01	N/A	N/A
N. m (L. c) 30 h, (n=3)	0	0.0	0.01	N/A	N/A

Figure 6B					
Sample	Average CFU count	Average CFU/ml x 10^3	std	P- value	Significance
L. c 6h, (n=3)	71	855	131	N/A	N/A
L. c (N. m) 6h, (n=3)	43	1418	443	N/A	N/A
L. c 22 h, (n=3)	89	129	4	N/A	N/A
L. c (N. m) 22 h, (n=3)	64	177	22	N/A	N/A
L. c 30 h, (n=3)	69	110	28	N/A	N/A
L. c (N. m) 30 h, (n=3)	55	139	39	N/A	N/A

Figure 6F					
Sample	Average CFU count	Average CFU/ml x 10^5	std	P- value	Significance
N. m	89	153	84		
N. m (L. c)	305	6.1	3.2	0.001	***
L. c	0	0.0	0.0	N/A	N/A
L. c (N. m)	0	0.0	0.0	N/A	N/A

REFERENCES

59. Sanders, H.; Brehony, C.; Maiden, M.C.J.; Vipond, C.; Feavers, I.M. The effect of iron availability on transcription of the Neisseria meningitidis fHbp gene varies among clonal complexes. *Microbiology (Reading, England)* **2012**, *158*, 869-876, doi:10.1099/mic.0.054957-0.
60. Sigurlásdóttir, S.; Wassing, G.M.; Zuo, F.; Arts, M.; Jonsson, A.B. Deletion of D-Lactate Dehydrogenase A in Neisseria meningitidis Promotes Biofilm Formation Through Increased Autolysis and Extracellular DNA Release. *Frontiers in microbiology* **2019**, *10*, 422, doi:10.3389/fmicb.2019.00422.
61. Sigurlasdottir, S.; Engman, J.; Eriksson, O.S.; Saroj, S.D.; Zguna, N.; Lloris-Garcera, P.; Ilag, L.L.; Jonsson, A.B. Host cell-derived lactate functions as an effector molecule in Neisseria meningitidis microcolony dispersal. *PLoS Pathog* **2017**, *13*, e1006251, doi:10.1371/journal.ppat.1006251.
